# Prevention of ureteral injury during laparoscopic colorectal cancer surgery with horseshoe kidney using fluorescent ureteral catheters: a case report

**DOI:** 10.1186/s40792-023-01604-z

**Published:** 2023-02-13

**Authors:** Tadahiro Kojima, Kiyotaka Kurachi, Kyota Tatsuta, Kosuke Sugiyama, Toshiya Akai, Katsunori Suzuki, Kakeru Torii, Mayu Sakata, Yoshifumi Morita, Hirotoshi Kikuchi, Yoshihiro Hiramatsu, Atsuko Fukazawa, Hiroya Takeuchi

**Affiliations:** 1 Department of Surgery, Hamamatsu University School of Medicine, 1-20-1 Handayama, Higashi-Ku, Hamamatsu, Shizuoka 431-3192 Japan; 2 Department of Perioperative Functioning Care and Support, Hamamatsu University School of Medicine, 1-20-1 Handayama, Higashi-Ku, Hamamatsu, Shizuoka 431-3192 Japan; 3 Department of Gastroenterological Surgery, Iwata City Hospital, 512-3 Ohkubo, Iwata, Shizuoka 438-8550 Japan

**Keywords:** Horseshoe kidney, Fluorescent ureteral catheters, Colorectal cancer

## Abstract

**Background:**

Horseshoe kidney is one of the most common congenital renal fusion anomalies and is characterized by abnormalities in the position, rotation, vascular supply, and ureteral anatomy of the kidney. When performing surgery for colorectal cancer in patients with horseshoe kidneys, anatomical identification is important to avoid organ injuries. Several reports on surgery for colorectal cancer with horseshoe kidneys have described the usefulness of three-dimensional (3D) computed tomography (CT) angiography for detecting abnormalities in vascular supply. However, few reports have focused on the prevention of ureteral injury in surgery for colorectal cancer with horseshoe kidney, despite abnormalities in the ureteral anatomy. Here, we report a case in which laparoscopic sigmoid colon resection for sigmoid colon cancer with a horseshoe kidney was safely performed using fluorescent ureteral catheters.

**Case presentation:**

A 60-year-old Japanese man presented to our hospital testing positive for fecal occult blood. Colonoscopy revealed sigmoid colon cancer, and CT confirmed a horseshoe kidney. The 3D-CT angiography showed aberrant renal arteries from the aorta and right common iliac artery, and the left ureter passed across the front of the renal isthmus. A fluorescent ureteral catheter was placed in the left ureter before the surgery to prevent ureteral injury. Laparoscopic sigmoid colon resection with D3 lymph node dissection was performed. The fluorescent ureteral catheter enabled the identification of the left ureter that passed across the front of the renal isthmus and the safe mobilization of the descending and sigmoid colon from the retroperitoneum. The operative time was 214 min, with intraoperative bleeding of 25 mL. The patient’s postoperative course was good: no complications arose and she was discharged on the seventh postoperative day.

**Conclusion:**

In patients with horseshoe kidney, the use of fluorescent ureteral catheters and 3D-CT angiography enables safer laparoscopic surgery for colorectal cancer. We recommend the placement of fluorescent ureteral catheters in such surgeries to prevent ureteral injury.

## Background

Horseshoe kidney—one of the most common congenital renal fusion anomalies—is characterized by abnormalities of the kidney that relate to its position, rotation, vascular supply, and ureteral anatomy [1–3]. Colorectal cancer surgery in patients with horseshoe kidney requires accurate anatomical identification to avoid injuries to organs such as the vessels and ureters. The risk of ureteral injury is higher in laparoscopic surgery than in open surgery because of the difficulty in identifying the ureter by palpation [4–7]. Several studies have reported that fluorescent ureteral catheters guide the identification of the ureter in real-time and reduce the risk of ureteral injury during surgery [8–10]. However, no reports have been published regarding performing surgery for colorectal cancer with a horseshoe kidney using intraoperative navigation with fluorescent ureteral catheters. We report a case in which laparoscopic sigmoid colon resection for sigmoid colon cancer with a horseshoe kidney was safely performed using fluorescent ureteral catheters.

## Case presentation

**Fig. 1:**
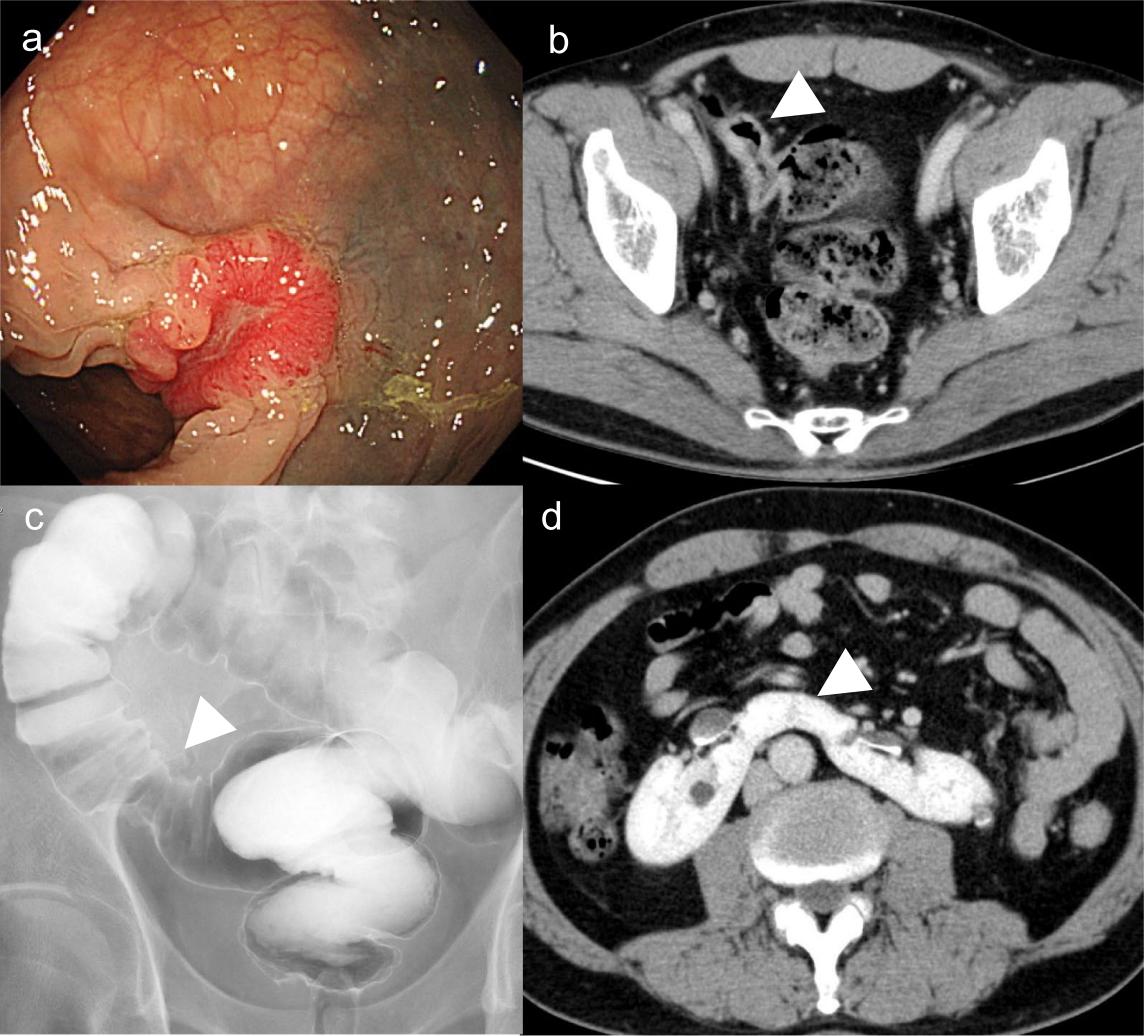
Preoperative findings. **a** Colonoscopy revealed a type-2 tumor in the sigmoid colon. **b** A contrast-enhanced computed tomography revealed sigmoid colon cancer (white arrowhead). **c** A contrast-enema revealed sigmoid colon cancer (white arrowhead). **d** The isthmus of the horseshoe kidney (white arrowhead)

**Fig. 2:**
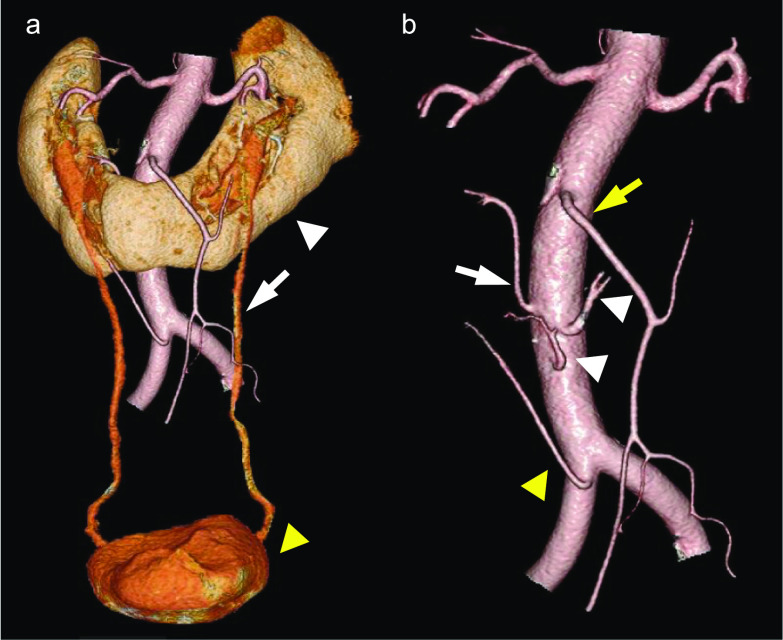
Three-dimensional computed tomography imaging showing the horseshoe kidney and aberrant renal arteries. **a** The horseshoe kidney (white arrowhead), left ureter (white arrow), and bladder (yellow arrowhead). **b** The root of the aberrant renal arteries at the isthmus from the aorta (white arrowhead), right branch of the aberrant renal artery from the aorta (white arrow), right branch of the aberrant renal artery from the right common iliac artery (yellow arrowhead), and inferior mesenteric artery (yellow arrow)

A 60-year-old Japanese man (weight 78.0 kg; height 179 cm; body mass index 24.3 kg/m^2^) presented to our hospital testing positive for the fecal occult blood. The patient had hypertension (American Society of Anesthesiologists physical status: class II). Sigmoid colon cancer was diagnosed using colonoscopy and contrast-enema, and a horseshoe kidney was confirmed by computed tomography (CT) (Fig. 1). Three-dimensional (3D) CT angiography showed aberrant renal arteries from the aorta and right common iliac artery, and the left ureter passed across the front of the renal isthmus (Fig. 2). To reduce the risk for intraoperative ureteral injury, a urologist placed the fluorescent ureteral catheter (IRIS U-Kits; Stryker, Tokyo, Japan) in the left ureter under general anesthesia before colorectal cancer surgery. The procedure required 5 min for completion, and no complications were noted.

**Fig. 3:**
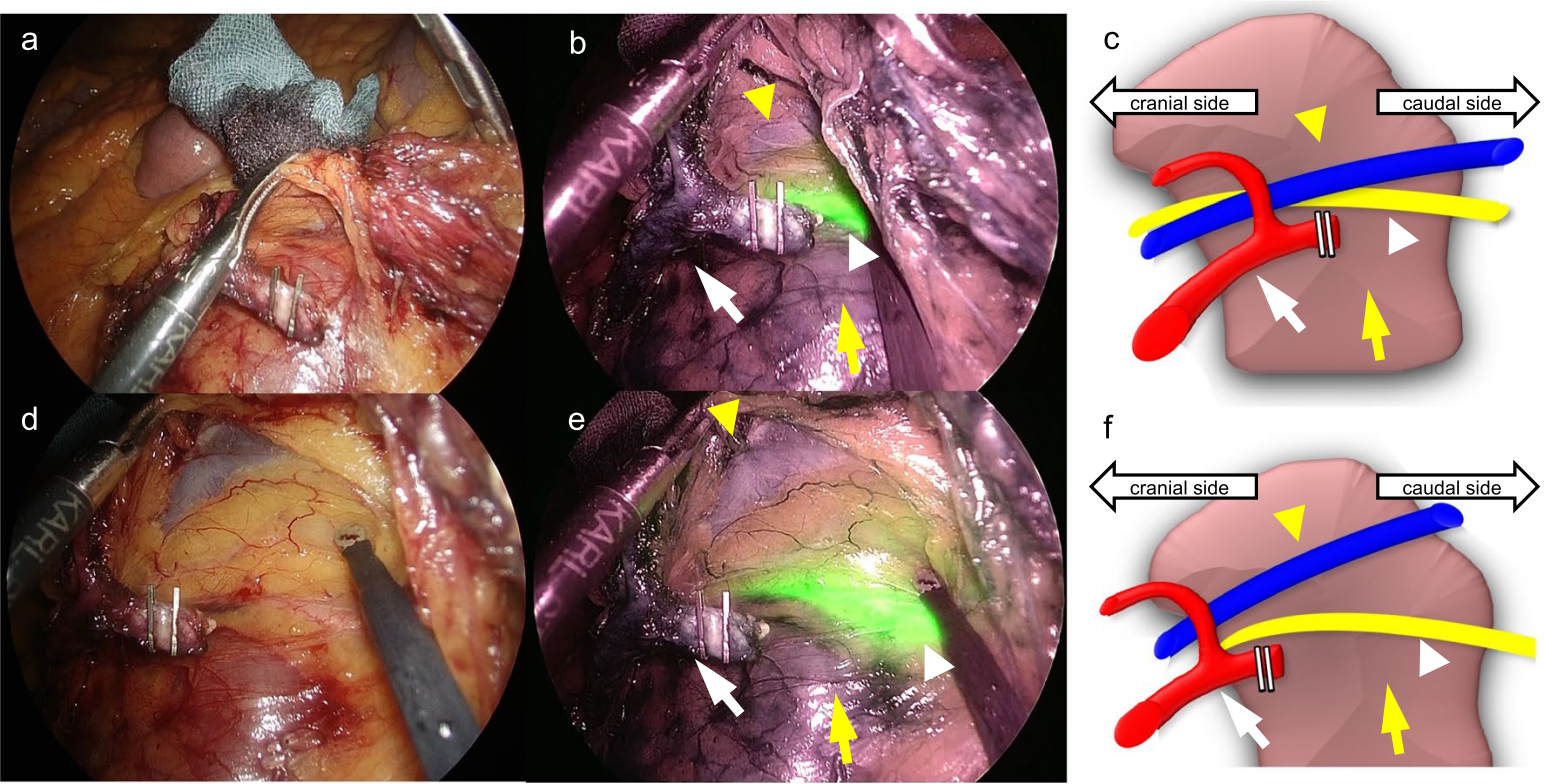
Intraoperative findings showing the ability of using a fluorescent ureteral catheter to visualize the ureter. **a** A white light image in the beginning of mobilizing the descending and sigmoid colons via a medial approach: the location of the left ureter is unclear. **b** A near-infrared light image in the beginning of mobilizing the descending and sigmoid colons via a medial approach: the left ureter was identifiable using the fluorescent ureteral catheter. **c** A schema in the beginning of mobilizing the descending and sigmoid colons via a medial approach.** d** A white light image during mobilizing the descending and sigmoid colons via a medial approach. **e** A near-infrared light image during mobilizing the descending and sigmoid colons via a medial approach. **f** A schema during mobilizing the descending and sigmoid colons via a medial approach. The left ureter (white arrowhead), inferior mesenteric artery (white arrow), inferior mesenteric vein (yellow arrowhead), and renal isthmus (yellow arrow) are shown

**Fig. 4:**
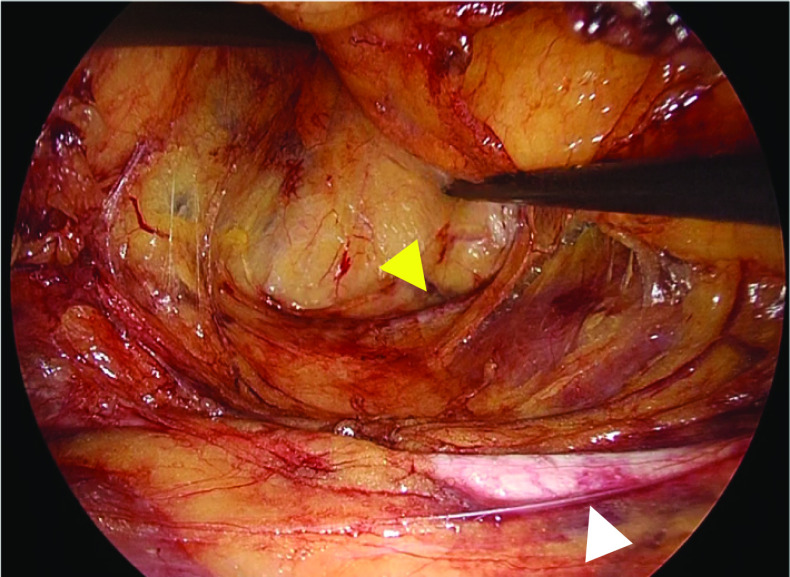
The relationship between the left ureter and left gonadal vessels. The left gonadal vessels ran lateral to the left ureter in mobilizing the descending and sigmoid colons via a medial approach. The left ureter (white arrowhead) and left gonadal vessels (yellow arrowhead) are shown

We performed a laparoscopic sigmoid colon resection with D3 lymph node dissection [11] using a five-port conventional technique, mobilizing the descending and sigmoid colons via a medial approach. The renal isthmus and aberrant renal arteries did not disturb the lymph node dissection for the root of inferior mesenteric artery and the dissection of vessels in this case. We used a laparoscopic scope (WAIR130A; Olympus, Tokyo, Japan), video system center (OLYMPUS OTV-300; Olympus, Tokyo, Japan), and light source (VISERA ELITE II XENON LIGHT SOURCE CLV-S200-IR; Olympus, Tokyo, Japan) to observe the fluorescent ureteral catheter in the near-infrared light. Using the fluorescent ureteral catheter, we were able to identify the left ureter that passed across the front of the renal isthmus and safely mobilize the descending and sigmoid colon from the retroperitoneum (Fig. 3). There was no anomaly in the relationship between the left ureter and left gonadal vessels; the left gonadal vessels ran lateral to the left ureter (Fig. 4). We could repeatedly identify the ureter easily during surgery by simply changing the white light images to near-infrared light images in the laparoscope. The operative time was 214 min, with intraoperative bleeding of 25 mL. The patient’s postoperative course was good and she did not experience any complications; she was discharged on the seventh postoperative day.

**Fig. 5:**
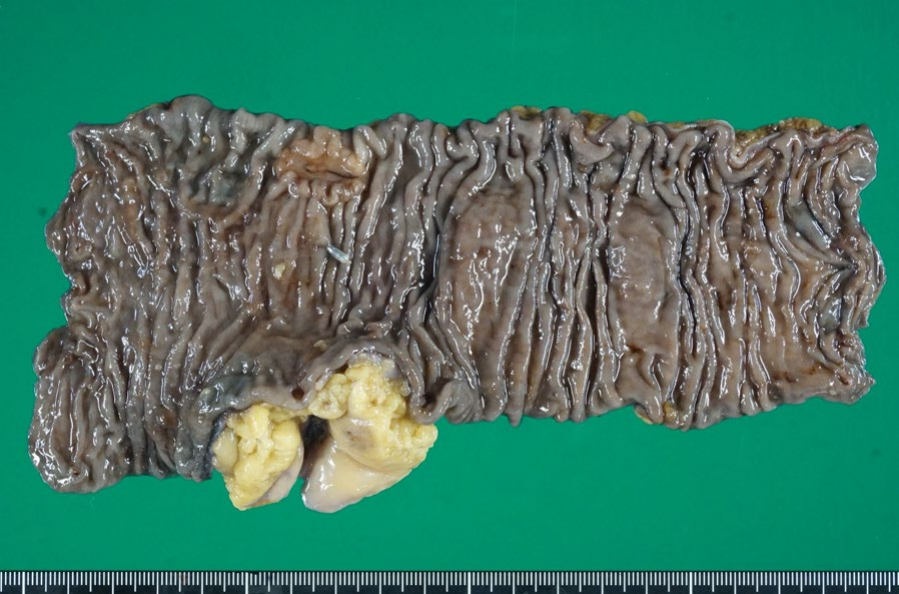
Macroscopic findings of the primary sigmoid colon cancer. A type-2 tumor (20 × 16 mm) was seen in the resected sigmoid colon and rectum

According to the 8th edition of the TNM classification [12], the pathological stage of the cancer was T2N1bM0 stage IIIA (Fig. 5). Ten months after the surgery, the patient is alive without recurrence.

## Discussion

The horseshoe kidney is a renal fusion anomaly that occurs during embryogenesis [1, 2]. The reported incidence of horseshoe kidneys is approximately 0.25% of the population [1]. When surgery is performed for colorectal cancer in patients with horseshoe kidneys, anomalous locations of the renal artery and ureter should be sufficiently evaluated to avoid iatrogenic injury. We report the usefulness of using fluorescent ureteral catheters in a case of laparoscopic surgery for colorectal cancer with a horseshoe kidney.

In the horseshoe kidney, the ureters frequently pass across the front of the renal isthmus. As the ureters in the horseshoe kidney are located more ventrally than those in the normal anatomy due to the renal isthmus, surgeons need to pay attention to iatrogenic injury. Furthermore, there are other ureteral anomalies, including ureters that pass behind the renal isthmus, arise from the renal isthmus, or ectopically open in regions other than the urinary bladder, as well as one ureter passing behind and the other in front of the renal isthmus, and duplication of the ureters [3]. Therefore, there is a high risk for ureteral injury and misidentification of anatomy in colectomy. In laparoscopic colectomy, dissection of the correct layer during mobilization of the mesentery prevents injury to the ureter, which is a retroperitoneal organ [13]. However, surgeons often dissect the retroperitoneal space in case of local advanced colorectal cancer requiring surgical margins or obesity, which makes identifying the correct layer difficult. Although in horseshoe kidney cases, the ureter is more likely to appear in the operating field because of a more ventral location than that in the normal anatomy, few reports have focused on how to prevent ureteral injury during laparoscopic surgery for colorectal cancer.

In abdominal surgery, the incidence of iatrogenic ureteral injury is estimated to be between 0.3% and 1.5% [14]. Risk factors for ureteral injury included a history of pelvic surgery, inflammatory bowel disease, endometriosis, and congenital renal abnormalities [6, 7, 10]. Ureteral catheter placement is a procedure used to prevent ureteral injury in such cases. In open surgery, placing a ureteral catheter helps in identifying the ureter by palpation. Contrarily, in laparoscopic surgery, it is difficult to visually identify normal ureteral catheters. However, fluorescent ureteral catheters can guide the visual identification of the ureter in real-time even during laparoscopic surgery, which is a significant advantage over imaging modalities such as CT. The use of fluorescent ureteral catheters has enabled the ureter to be safely preserved in laparoscopic surgery even for cases of severe adhesions in diverticulitis or colorectal cancer surgery after total cystectomy [9, 10]. In recent years, robotic abdominal surgery has become widespread. However, in robotic surgery, the operator has no sense of touch at all. Thus, visual information is more crucial; consequently, the use of fluorescent ureteral catheters is more effective in robotic surgery. A previous study reported that the ureter could be identified in 91.3% of cases using fluorescent ureteral catheters during colorectal surgery [8]. Furthermore, the identification of the fluorescent ureteral catheters that use near-infrared light is simple and easy with only the mode conversion of the laparoscope. In cases where more time is required to identify the ureter during surgery, visualization of the ureter using fluorescent ureteral catheters can help shorten the operative time. In addition, the fluorescent ureteral catheters that use near-infrared light as in this case do not generate heat; therefore, there is less risk for thermal damage to the ureter. In this case, we could perform surgery more safely and efficiently by using the fluorescent ureteral catheter.

Many reports on surgery for colorectal cancer cases with horseshoe kidneys have described the usefulness of 3D-CT angiography to detect abnormalities in vascular supply [15–18]. Furthermore, the use of fluorescent ureteral catheters to detect abnormalities in the ureteral anatomy has enabled laparoscopic surgery to be performed more safely, as in this case. In laparoscopic surgery cases with ureteral anomalies, such as horseshoe kidneys, we highly recommend placing fluorescent ureteral catheters to prevent ureteral injury.

## Conclusion

Laparoscopic surgery for colorectal cancer in a patient with a horseshoe kidney carries a risk for ureteral injury. This risk can be reduced by using fluorescent ureteral catheters to identify the ureters.

## Data Availability

Data sharing is not applicable to this article, as no datasets were generated or analyzed during the study.

## Abbreviations

CT: Computed tomography
3D: Three-dimensional
